# Teachers' expertise in feedback application adapted to the phases of the learning process

**DOI:** 10.3389/fpsyg.2014.00858

**Published:** 2014-08-05

**Authors:** Eva Christophel, Robert Gaschler, Wolfgang Schnotz

**Affiliations:** Department of Psychology, Universität Koblenz-LandauLandau, Germany

**Keywords:** teacher expertise, motivational phases, learning phases, feedback, curriculum scripts

## Building better brains—school day by school day

In this essay we highlight feedback application as a domain to study the knowledge and abilities involved in the construct of teachers' expertise. One approach to advance the discussion in a field of research such as expertise acquisition generally is to combine (a) analysis of complex natural phenomena by division into *hypothetical* simple building blocks, with (b) the synthesis of complex phenomena based on *known* simple building blocks (e.g., Braitenberg, 1984; Gaschler et al., 2012). For instance, (a) knowledge representations such as chunks have been hypothesized to underlie chess expertise and (b) this analysis in turn was supported by a computer-run expert system based on chunks (cf. Lane et al., 2001; Guida et al., 2012). Gobet et al. (2014) summarize research on expert knowledge as well as the Einstellung effect (e.g., missing a to spot an efficient procedure, because a well-known one is available; cf. Bilalić et al., 2010), and neuro-interventions targeting it. They conclude with the desideratum to build better brains—brains that can take full advantage of the power of hypothesis-driven cognition while being safeguarded against cognitive illusions and the Einstellung effect.

In the current article we argue that building better brains is (an admittedly to-be-optimized) everyday practice, rather than a thought experiment. Like Gobet et al. (2014) we ask how creativity can be fostered by gaining control over prior knowledge so that it can be flexibly used or blocked at demand (cf. Bilalić et al., 2008). Teacher education in universities delivers quasi-experimental conditions for studying how expertise on learning can be acquired and applied best. In particular, concepts of motivation and action control relevant in robotics and psychology seem promising in order to capture and structure the gist of teacher expertise.

## Becoming experts in facilitating knowledge acquisition

While expertise is often studied in domains that yield only hundreds or thousands of experts, universities all over the world are taking efforts to continuously contribute to a large population of experts on knowledge acquisition. Based on theoretical input and years of practice, teachers should be experts for shaping school lessons such that knowledge acquisition is optimized (Bromme, 1997, 2008). Focusing on the demands placed by school lessons can help to obtain an overview on the skills teaching experts should have (Bromme, 2008). Teachers have to organize and maintain a structure of student and teacher activities. This includes the anticipation of students' inferences as well as disturbances of the social context of learning. Teachers need a broad and flexible knowledge base covering the subject, as well as a repertoire of instructional methods to stimulate students' learning activities for reaching instructional objectives. Like managers, teachers have to organize the timetable of each lesson to make sure that the time is mainly used for the subject matter. Shulman (1986, 1987) attributes to teaching experts a repertoire of content knowledge (e.g., diagnosis of task specific requirements), pedagogical content knowledge (e.g., how to present the subject matter content), and curricular knowledge. Leading to the appropriate generation and scheduling of feedback to the students, teaching experts should have a strong background in the philosophy of the subject (e.g., subject-related epistemic beliefs), diagnostic competences (e.g., judgment of students' abilities), and skills that allow them to juggle between student-related aspects of the learning situation as well as content-related aspects (Bromme, 2008). Teachers' instructional routines include categorical units called “curriculum scripts,” in which subject-related and didactic-methodological aspects are linked. As a consequence of this integrated knowledge, most actions of experienced teachers proceed automatically (Blömeke et al., 2003). They are difficult to access via verbal protocols, because in the classroom or a face-to-face situation, verbalization of the teacher could reduce the learners' amount of cognitive resources and shift the learner's attention to the level of meta-task processes.

## Feedback aligned to motivational and volitional states of action regulation

Learners face the challenge to acquire and employ self-regulation strategies in order to obtain educational outcomes (e.g., Lerner et al., 2001; Ley and Young, 2001). In order to scaffold learning through adaptive feedback, teachers need knowledge about the dynamics of learning processes and opportunities to apply feedback that takes motivational, cognitive and emotional aspects of learning into account. Apart from finding the appropriate frequency for feedback (Healy et al., 2014), we suggest consideration of the match of feedback type and action phase the learner is currently in Heckhausen and Gollwitzer (1987) have proposed distinguishing between motivational and volitional phases of action regulation. The distinction between motivational and volitional phases of action regulation has been used in two different ways to bridge the gap between analysis and synthesis highlighted by Braitenberg (1984). On the one hand, many architectures of artificial agents in robotics implement the distinction between a motivational phase (the agent is open to new information and ready to re-evaluate current preferences and plans) and a volitional phase (the agent is executing a plan and shielding itself from novel information that might lead to a re-evaluation of the plan currently executed; e.g., Visser and Burkhard, 2007). On the other hand, cycles of motivational and volitional phases are present in theories of the cyclical and recursive dynamic of learning in educational contexts (e.g., Zimmerman, 2000; Schmitz and Wiese, 2006).

According to Zimmerman (2000) as well as Schmitz and Wiese (2006), the learning process proceeds in three phases. (1) In the goal-setting phase, the learner chooses goals (e.g., appropriate tasks), plans actions or action steps and selects adequate strategies. (2) In the performance phase, the selected strategies have to be applied in order to complete the task. (3) In the self-reflection phase, the learner evaluates his/her learning outcome. Gollwitzer (1990) assumes that in each one of these phases the learners' attention is focused on specific information that helps him/her to accomplish the demands of the task. Taking the current focus of the learner into account, feedback can be better adapted with respect to effects on the learners' performance, mood and effort (Ley and Young, 2001; Narciss, 2004; Baadte and Schnotz, 2013).

Three types of feedback can be distinguished that align to the respective phases of the learning process. In the goal setting phase, the learner should receive goal-setting feedback that informs him/her about how realistic completion of the chosen task is according to his/her previous performances. In the performance phase, process-feedback offers information about specific task-inherent demands and error-related information. In the self-reflection phase, the learner should obtain appropriate outcome-feedback that informs him/her about the possible causes for success or failure and about the quality of his/her performance. In contrast, feedback is inappropriate if it does not take the learner's phase-specific mind-set into account and if it does not support the task completion but instead decreases the learner's amount of cognitive resources available for processing the relevant information (e.g., Sweller, 2005; see Christophel and Baadte, in press).

Matching feedback to the phase-specific requirements of the learning process might require flexible strategy changes and adaptation of routines to the specific content being taught, epistemic beliefs and motivational/volitional state diagnosed. Thus, adaptation and shifting skills seem at least as important as a large repertoire of strategies. This view is reflected in the notion of adaptive expertise advocated in education psychology (Verschaffel et al., 2009; Godau et al., 2014). In contrast to routine expertise (e.g., expertise that allows the expert to solve problems very efficiently and precisely), Hatano and Inagaki (1984) describe adaptive expertise as the potential to create new solutions and new problem solving procedures. Adaptive experts are “those who not only perform procedural skills efficiently but also understand the meaning of the skills and nature of their object” (p. 28). In contrast, “routine experts are outstanding in terms of speed, accuracy, and automaticity of performance, but lack flexibility and adaptability to new problems” (Hatano and Inagaki, 1984, p. 31). Verschaffel et al. (2009) have pointed out that *flexibility* can be defined as the “use of *multiple* strategies” while *adaptivity* includes “making *appropriate* strategy choices” (p. 338). This flexibility and adaptivity is necessary in order to support students' individualized learning processes with appropriate feedback. Thus, teachers need adaptive expertise which should include knowledge about the task specific requirements, the learner abilities, the cyclical and recursive dynamic of the learning process and the implications of this dynamic on motivational, cognitive and emotional levels. On the one hand, there are first formalized efforts to teach future teachers relevant motivational competencies (e.g., Rheinberg and Engeser, 2010). On the other hand, there are more cautious outlooks as well. According to Verschaffel et al. (2009) “adaptive expertise is not something that can be *trained* or *taught* but rather something that has to be *promoted* or *cultivated*” (p. 348).

## More teaching experience does *not* necessarily lead to better feedback skills

Teachers' expertise emerges during the theoretical and practical phases of teacher education and professional experience after university education (Bromme, 2008). It is an open question as to what extent the professional experience of teachers promotes and cultivates adaptive expertise with regard to adequate students' support in individualized lessons. For instance, Christophel (2014) demonstrated that more experienced teachers did *not* give more appropriate feedback (i.e., feedback that offers phase-specific information) but actually gave more inappropriate feedback than less experienced teachers (e.g., feedback that does not support the task completion but reduces the learner's amount of cognitive resources available for processing the relevant information; Sweller, 2005). Christophel (2014) studied 30 more experienced teachers with a mean professional experience of 11.15 years (*SD* = 12.85) and 30 less experienced teachers with a mean professional experience of 42.07 days (*SD* = 89.40). Teachers watched three video-vignettes of students passing through the different phases of self-regulated learning (goal-setting, performance and the self-reflection phase). The teachers were instructed to stop the films whenever they wanted to give feedback to the students. The results revealed that the more experienced teachers stopped the video-vignettes more frequently and more often provided inappropriate feedback to students as compared to their less experienced counterparts. In addition, the study of Baer et al. (2011) showed that professional experience did not lead to better schooling. Baer and colleagues examined the development of teacher competences in transition between academic and professional careers. The results demonstrate that teachers with more professional experience did not perform better in the measured aspects of schooling than graduate teachers at the end of their university education.

These findings suggest that professional experience alone is not a good predictor for the progress of teachers' expertise (Baer et al., 2011; cf. Campitelli and Gobet, 2011; Hambrick et al., 2014). Years of teaching are possibly not sufficient to informally and implicitly sensitize most teachers to cues conveying action phases and the matching feedback. This could have diverse reasons. As explained above, school lessons place numerous demands: teachers have to organize and maintain the structure of schooling and can be absorbed by organizational and administrative activities. After university and practical education, there is a lack of peer-coaching (e.g., colleagues who discuss classroom situations) or qualified instruction (e.g., best-practice examples). A lack of (time) resources and motivation to reflect ones feedback behavior might result.

## Attending the larger picture

However, training studies can support an optimistic outlook on the development of teachers' expertise in feedback application. Experienced and inexperienced teachers can be trained to apply feedback that supports realistic goal-setting and adequate self-reflection of learners (Christophel et al., in press). Recommendations helped teachers to increase feedback in line with the motivational phase of the learner from pre- to post-test while phase-inappropriate feedback could be reduced. Also, attention allocation in the classroom—often a prerequisite for feedback application—seems to be open to intervention. For instance, Miller (2011; cf. Speelman, 2014; Wiggins et al., 2014) suggested to employ eye-tracking in order to provide (becoming) teachers with feedback on how evenly they distribute their attention across students in the classroom. Students disturbing the setting should not monopolize attention at the cost of other students. While experienced teachers agree that uneven distributions of attention should be avoided, they lack awareness of how well they are yet managing to implement the respective strategies of attention allocation.

### Conflict of interest statement

The authors declare that the research was conducted in the absence of any commercial or financial relationships that could be construed as a potential conflict of interest.
